# Factors Influencing Viral Clearance in Mild COVID-19 and Clinical Characteristics of Asymptomatic Patients

**DOI:** 10.1155/2021/5909612

**Published:** 2021-02-26

**Authors:** Hong-mei Shu, Shi He, Yong Sun, Chang-qing Lin, Yu-fang Lu, Jing Liu, Ting Wu, Li Li, Guo-zheng Ding, Wei Shi, Zhen-jun Liu

**Affiliations:** ^1^Department of Respiration, Anqing Municipal Hospital, Anhui, China; ^2^Infection Disease Department, Anqing Municipal Hospital, Anhui, China

## Abstract

**Background:**

The outbreak of coronavirus disease (COVID-19) has become a global public health emergency.

**Objective:**

To evaluate the characteristics and outcomes of patients with COVID-19 in Anhui and to identify predictors of viral clearance.

**Methods:**

We retrospectively analyzed the data collected from discharged patients with laboratory-confirmed SARS-CoV-2 infections. We compared clinical features between viral clearance and viral persistence, and evaluated factors associated with SARS-CoV-2 shedding using multiple linear regression.

**Results:**

Among the 83 patients involved in the study, the median age was 43 years, while 60.2% were male, 35.4% had comorbidities, and the mortality was zero. The median time from illness onset to admission was 5 days (interquartile range (IQR), 2-7 days), and the median time from the illness onset to SARS-CoV-2 RNA detection was 16 days (IQR, 13-18 days). The factors influencing viral clearance were as follows: (1) delayed admission (beta 1.057, 95% CI 0.810-1.304; *p* ≤ 0.001) and (2) underlying comorbidities (beta 1.907, 95% CI 0.198-3.616; *p* = 0.029). No significant differences were observed in the length of stay (*p* = 0.246) and pneumonia between asymptomatic and symptomatic patients based on computed tomography (CT) (*p* = 0.124).

**Conclusions:**

Delayed admission and underlying comorbidities may effectively predict SARS-CoV-2 RNA clearance. For those infected with SARS-CoV-2, even asymptomatic patients without any clinical symptoms should be traced and isolated. This practice may reduce the spread of SARS-CoV-2 and slow the COVID-19 pandemic caused by the virus. *Clinical Trial Registration Number*: This trial is registered with 2020-051.

## 1. Introduction

In December of 2019, Wuhan City in Hubei Province of China became the center of an outbreak of pneumonia of an unknown origin. A novel coronavirus (CoV) was soon isolated from patients in Wuhan [1, 2]. This virus, SARS-CoV-2 [3], has subsequently spread across the world, and the disease it caused was named as the coronavirus disease 2019 (COVID-19) in February 2020 by the World Health Organization (WHO) [4]. As of 27 April 2020, the WHO has notified of 3 million laboratory-confirmed infections with SARS-CoV-2 in 209 countries; the resultant COVID-19 disease has been labeled a Public Health Emergency of International Concern by the WHO [5].

A great deal of evidence for the human-to-human transmission of COVID-19 has been reported by previous papers. For example, Huang et al. [6] first reported 41 cases of COVID-19 with clinical characteristics including fever, nonproductive cough, dyspnea, myalgia, and fatigue, while Zhou et al. [7] reported a number of other potential risk factors including older age, high Sequential Organ Failure Assessment (SOFA) score, and D-dimer for adult inpatient mortality for COVID-19 in Wuhan. High titers of viral RNA were detected soon after the symptom onset, while symptomatic and asymptomatic patients had similar viral loads according to a further study [8].

While the published findings focused on critical illnesses, patients with mild symptoms, which account for the majority of infections, were reported very rarely. Relatively mild symptoms for patients in Zhejiang Province and no mortalities were reported by Xu et al. [9]. Thus, it is essential to notify the asymptomatic cases or the cases with mild symptoms. We analyzed retrospectively collected data for 83 patients with laboratory-confirmed SARS-CoV-2 infections, who were admitted to Anqing Municipal Hospital in Anhui Province. In this manner, we aimed to explore the key factors involved in the duration of viral clearance in patients and compare the outcomes between symptomatic patients and asymptomatic carriers.

## 2. Materials and Methods

### 2.1. Study Design and Participants

The single-center retrospective observational study was conducted in Anqing Municipal Hospital, Anhui Province, China. Information on all discharged patients was collected from this hospital, which is specialized in infectious diseases. This case series was approved by the Institutional Ethics Board of Anqing Municipal Hospital (no. 2020051).

### 2.2. Definitions

The date of diagnosis was defined as the day when the first sample tested positive for SARS-CoV-2 by qRT-PCR. Criteria for discharged patients were defined as consecutive negative tests after every other day through throat swabs and/or stool swabs. The cessation of viral clearance was concluded by the first negative qRT-PCR with no subsequent positive swab. The median duration of SARS-CoV-2 detection was 16 days. Patients were further divided into two groups: one group involved patients with persistent negative viral detection results < 16 days after the illness onset (*n* = 35) (defined as viral clearance) and the other group had patients with prolonged viral RNA shedding ≥ 16 days after the illness onset (*n* = 36) (defined as viral persistence) [10, 11]. A total of 71 patients were included in this cohort analysis. Specimens of throat swabs and/or stool swabs were collected every other day following admission. Fever was defined as an axillary temperature of at least 37.3°C. Asymptomatic infection was defined as any patient testing positive for SARS-CoV-2 by close contact, but presenting no clinical symptoms. Comorbidities were defined as preexisting underlying diseases.

### 2.3. Covariates

The candidate variables considered for the analysis of prolonged duration of SARS-CoV-2 shedding were as follows: gender (male/female), age, BMI (18.5–24 = 0, <18.5, or >24 = 1), comorbidities (no/yes), corticosteroid (no/yes), pulmonary infiltration (no/yes), and time from the illness onset to admission.

### 2.4. Procedures

Epidemiological, demographic, clinical, laboratory, treatment, and outcome data were obtained from electronic medical records, all reviewed by specialized physicians.

The presence of SARS-CoV-2 was confirmed using real-time qRT-PCR by the same protocol described previously in a study by the Anqing Centers for Disease Control and Prevention [12]. The detection reagents used in the present study were provided by Shanghai BioGerm, Shanghai GeneoDx, and Guangzhou DaAn institution, all approved by the provincial Centers for Disease Control and Prevention (CDC). Throat swabs and/or stool swabs (after 11 February) were taken for SARS-CoV-2 PCR reexamination from all patients every other day starting at admission, because SARS-CoV RNA was found in fecal samples of patients. In the meta-analysis [13], the pooled prevalence of stool samples positive for viral RNA was 48.1% (95% CI, 38.3%–57.9%); 70.3% of such samples collected after the loss of virus from respiratory specimens were positive (95% CI, 49.6%–85.1%). Routine laboratory tests, including hematology, biochemistry, radiology, and microbiological investigations, were conducted in the laboratory, and chest CT was performed for all inpatients. The frequency of these examinations was determined by the treating physician at the hospital.

### 2.5. Outcomes

Clinical data were presented, including demographics, epidemiology (i.e., family cluster and contacts from Wuhan), comorbidity, signs and symptoms on admission, dynamic test results, chest radiography, and CT findings, as well as treatments received for COVID-19.

### 2.6. Statistical Analysis

All analyses were performed using the software SPSS (version 22.0). Continuous and categorical variables were presented as median (interquartile range (IQR)) and *n* (%), respectively. Continuous variables forming a normal distribution were compared using *t*-tests, while those following nonnormal distributions were compared using the Mann-Whitney *U* test. Cases were compared with one another and with controls using Pearson's chi-squared test or Fisher's exact test, as appropriate for categorical variables. To explore the factors that influence viral clearance, multivariable linear regression models were used. Variables with *p* ≤ 0.05 in univariate models using stepwise analysis were candidates for multivariate models, where a two-sided *α* of less than 0.05 was considered statistically significant.

## 3. Results

### 3.1. Patient Demographic Data

A total of 83 cases were included in our research. One of these cases was severe, where the patient was transferred to Anhui Provincial Hospital after seven days of stay in our hospital; however, he was discharged after clinical follow-up. Mild symptoms were prevalent in 71 patients, while asymptomatic infection was present in 11 patients. The clinical mortality rate was zero (Table 1).

Of these patients, thirty-three patients included familial cluster as sources of infections. 11 cases of asymptomatic infection occurred due to close contact, while 85.5% of which are mild infection cases. The bulk of the patients were men, with a mean age of 43 years (IQR 33-53; Table 1), while 30 (36.1%) had comorbidities. The most common underlying diseases encountered were hypertension followed by chronic hepatitis and postoperative biliary disease, diabetes, and chronic lung disease (Table 1), respectively. At admission, most patients had fever (70.7%), fatigue (35.8%), chills (29.3%), dry cough (29.3%), and expectoration (28.0%). The comparison of age, BMI, and comorbidities between the viral clearance and persistence is shown in Table 1. The median age of the viral persistence cases (48.0 years) was significantly older than that of the viral clearance cases (37.0 years). Eight (22.9%) have comorbidities in the viral clearance group, while 17 (47.2%) have comorbidities in the other group.

### 3.2. Laboratory Findings

Hypoproteinemia occurred in 39 (47%) patients, and lymphopenia was below the normal range in a significant number of patients (Table 1). Levels of D-dimer and activated partial thromboplastin at the time of admission were above the normal range. C-reactive protein was above the normal range among 52 (63%) inpatients on admission. Data show that 10 (12%) of patients had a normal CT value. 33 (39.8%) patients had bilateral ground-glass shadows, while 25 (30.1%) patients had multiple patchy shadows (Table 1). The different sizes of patches with a clear or ground-glass edge have a sheet pattern.

### 3.3. Treatment Protocols and Clinical Outcomes

Chu et al. found that lopinavir/ritonavir treatment was associated with a better outcome even when adjusted for baseline lactate dehydrogenase levels in patients with SARS [14]. Given the lack of effective antiviral therapy against COVID-19, current treatments are mainly based on experience from fighting the previous epidemics of SARS-CoV and MERS-CoV [15]. Almost all patients received combination therapy according to national guidelines for the diagnosis and management of COVID-19 [16], which consisted of interferon (IFN) alpha-2b inhalation and lopinavir/ritonavir. A total of 80 (96.4%) patients received traditional Chinese medicine, and 81 (97.6%) were administered lopinavir/ritonavir. The duration of lopinavir/ritonavir therapy was 10 days (IQR 10-11). Nasal oxygen therapy was used in 46 (55.4%) patients, whereas systemic corticosteroids were applied in 23 patients of whom 12 (33.3%) showed prolonged viral clearance.

The median duration of viral clearance was 16 days (IQR 13-18). The observed length of viral shedding varied between 8 and 34 days. The time from the illness onset to admission was 5 days (IQR 2-7), while the length of diagnosis delay was 4.5 days (IQR 2-7). The median hospital stay was 16 days (IQR 14-19). Similarly, the median duration from illness to the confirmation of recovery was 15 days by chest radiography.

We included 71 mild patients with complete data for the study of all variables (35 viral clearance and 36 viral persistence cases). The laboratory results did not differ between the viral clearance and viral persistence groups. Baseline data showed older median age, underlying diseases, and lower BMI in the viral persistence group compared with the viral clearance group (Table 1). For the viral clearance group, the median time from the illness onset to admission was 3 days (IQR 2-5), while radiologic recovery was 11 days (IQR 9-15). For the viral persistence group, the median delayed admission and radiologic recovery were 7 days and 17 days, respectively (Table 2).

### 3.4. Relative Risk Factors for Prolonged Viral Clearance

In the univariate linear regression model, delayed admission and underlying comorbidities were associated with increased odds of viral persistence by stepwise analysis (Table 3). The currently available evidence is insufficient to determine the effectiveness of corticosteroids for people with influenza [17]. A rare case of human coronavirus 229E was associated with the acute respiratory distress syndrome [18]. Simon et al. [19] reported a case of pneumonia due to an infection with human coronaviruses- (HCoVs-) OC43 in a pediatric leukemia patient with Down syndrome and febrile neutropenia. Therefore, corticosteroids and pneumonia were forced into the multivariate linear regression model. There were significant associations among time from the illness onset to admission (*B* = 1.057, *b* = 0.746, *p* ≤ 0.001) and comorbidities (*B* = 1.907, *b* = 0.191, *p* = 0.029). (Table 3). Then, it showed the prolonged SRAS-CoV-2 RNA clearance increased by 1.057 days for each delayed admission day, and 1.907 days for each additional day with underlying comorbidities.

### 3.5. Asymptomatic and Symptomatic

**Figure 1:**
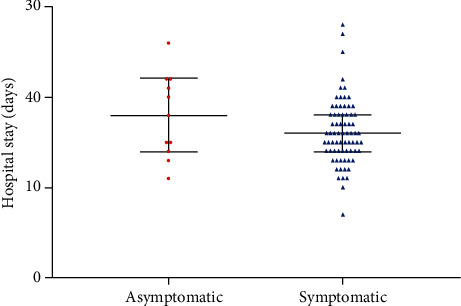
The median hospital stay of symptomatic patients and asymptomatic carriers was 16 days (IQR 14-18) and 18 days (IQR 14-22), respectively. No significant differences in the hospital stay between symptomatic and asymptomatic carriers were found.

**Figure 2:**
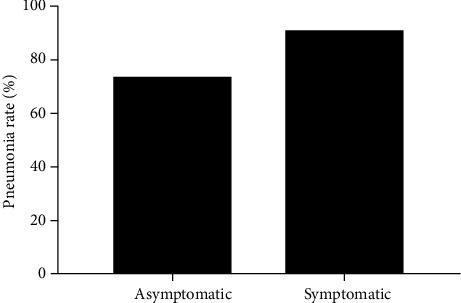
Fisher's exact test results between asymptomatic and symptomatic cases, with no differences found between the two groups for pneumonia.

Asymptomatic infection occurred with a mean age of 53 years (IQR 34-55, Table 1), while 72 (86.7%) symptomatic patients were 40 years (IQR 32.5-51.75) of median age. There were significant differences in levels of C-reactive protein, procalcitonin, and high-sensitivity cardiac troponin I between symptomatic and asymptomatic cases (Table 1). We further compared the length of stay between the asymptomatic and symptomatic groups using Mann-Whitney *U* tests. The median hospital stay was similar in symptomatic patients of 16.0 days (IQR 14.0-18.0) and in asymptomatic carriers of 18.0 days (IQR 14.0-22.0). There was no statistically significant difference between the two groups (*p* = 0.246) (Table 2, Figure 1). We also observed if pneumonia was different between symptomatic and asymptomatic groups. It was discovered that 8 asymptomatic patients had pneumonia while 7 symptomatic patients had normal chest CT. Interestingly, there was no difference in groups (*p* = 0.124) (Table 1, Figure 2).

## 4. Discussion

This retrospective cohort study identified a number of influencing factors for the length of viral clearance in COVID-19 patients and conducted different factors for predicting the influence of viral persistence. Our findings showed that of the delayed admissions, comorbidities appeared to be associated with viral clearance. Under the situation of lack of effective therapies to SARS-CoV-2 and the COVID-19 disease it causes, lopinavir/ritonavir and IFN a-2b were recommended to patients. There is no difference between the two groups in the baseline and treatment except for variables ages, BMI, and comorbidities.

Delayed admission was an independent influence factor associated with prolonged SARS-CoV-2 RNA clearance. COVID-19 patients had clinical manifestations of fever, cough, and fatigue, which are no different from other viral infections [5]. Thus, at early illness, they would ignore their symptoms and delay visiting a doctor. Indeed, the median duration of time from illness onset to admission was 5 days, whereas it has been reported 12 days by Zhou et al. [7]. Meanwhile, we detected that the median duration of viral clearance was 16 days. One recent report showed that the median duration of viral clearance in COVID-19 was 20 days in patients with severe illness and could be as long as 37 days [7]. Recent reports of 56 patients diagnosed with mild to moderate COVID-19 found that the median duration between the onset of symptoms and nucleic acid conversion was 24 days, and virus shedding was up to 6 weeks after the onset of symptoms [20]. This difference may also vary among patients. In severe influenza virus infection, prolonged clearance was associated with fatal outcomes and delayed antiviral treatment, an independent risk factor for prolonged virus detection [21]. Compared with the viral persistence group, patients with viral clearance may receive timely diagnosis and prompt treatment. Early admission and antiviral treatment were the key to ending early viral clearance and stopping the spread of virus.

In addition, patients with comorbidities showed prolonged viral RNA shedding. Hypertension was the most comorbidity in our research. This is consistent with most studies [10, 22]. A report of 416 hospitalized patients with COVID-19 concluded that cardiac injury is associated with a higher risk of in-hospital mortality [23]. Not only was COVID-19 96% identical at the whole-genome level to a bat coronavirus but also it is confirmed that it uses the same cell entry receptor, ACE2, as SARS-CoV [24]. Recently, it has been described that the disease's severity may depend on the size of the infectious viral inoculum and/or an individual's ability to clear the infection [25]. It has been reported that CHD (coronary heart disease) was proven to be an independent risk factor for prolonged viral RNA shedding [26].

There has been controversy regarding whether corticosteroid use may delay viral clearance in patients with viral pneumonia for a long time. A systematic review [27] reported on 15 studies, 13 of which were inconclusive to any benefits of corticosteroids. It is difficult to make a clear recommendation about whether corticosteroids should be used to treat SARS-associated lung injury at any stage of illness, particularly as these drugs are immunosuppressive and may delay viral clearance if administered before viral replication is controlled [28]. Wang et al. reported that corticosteroid therapy was associated with prolonged A(H7N9) RNA shedding [21]. One RCT reported that the delayed clearance of SARS-CoV-1 viral loads was associated with corticosteroid use [29].Our research found that corticosteroid therapy in patients has no effect on viral clearance in accordance with Ma's report [30].

Previous research has shown that pathogenic human coronavirus infections result in severe pneumonia. Also, SARS-CoV-2 infection induces acute viral interstitial pneumonia in rhesus macaques [31]. It was associated with rapid virus replication, massive inflammatory cell infiltration, and elevated proinflammatory cytokine/chemokine responses resulting in acute lung injury (ALI) and acute respiratory distress syndrome (ARDS) [32]. In contrast, MERS-CoV-infected rabbits displayed mild clinical disease with mild-moderate perivascular and peribronchiolar infiltration and, to a lesser extent, interstitial lung inflammation [33, 34].However, in this study, pulmonary infiltration was not associated with viral clearance.

The samples were taken by postmortem biopsy in asymptomatic patients with respect to SARS-CoV-2. The findings were nonspecific and included edema, pneumocyte hyperplasia, focal inflammation, and multinucleated giant cell formation, while no hyaline membranes were seen [35]. Interestingly, no difference in the length of stay and pneumonia was seen between asymptomatic and symptomatic patients. Long et al. [36] reported that in comparison to symptomatic patients, the asymptomatic group had a significantly longer duration of viral shedding, with a viral shedding time of 19 d. They concluded that asymptomatic individuals exhibited lower levels of 18 pro- and anti-inflammatory cytokines. This may explain the viral replication in asymptomatic carriers. Asymptomatic carriers with confirmed SARS-CoV-2 demonstrated significant pulmonary findings by CT screening. We found that the age was older in asymptomatic patients than in symptomatic patients. This is in accordance with our viral persistence in old age.

This study has several limitations. Firstly, not all laboratory tests were performed regularly because this was a retrospective study involving a single medical center. Second, viral mRNA was detected using a qualitative assay, and only a throat swab was tested at an early stage, which was then used for viral load calculation. Third, the interpretation of our findings might be limited by sample size. This result was derived for general patients, so whether it is applicable to patients with severe conditions is uncertain.

## 5. Conclusion

Previous studies have found that older age and underlying conditions were factors in predicting prolonged viral clearance in COVID-19 patients. Our study looked at not only underlying disease but also early admission and CT examination in patients with mild infections and asymptomatic carriers. This may help to confirm or exclude the possibility of diagnosis. Asymptomatic patients without any clinical symptoms (infected by close contact), who were confirmed by swab-throat RT-PCR and had abnormal chest CT images, should be traced and isolated.

## Figures and Tables

**Table 1:** Demographics and clinical characteristics of the patients at baseline.

	Total	Mildly symptomatic			Total confirmed SARS-COV-2		
		Viral clearance	Viral persistence	*p* value	Symptomatic	Asymptomatic	*p* value
	*n* = 83	*n* = 35	*n* = 36		*n* = 72	*n* = 11	
Age (years)	43 (33-53)	37 (28-46)	48 (37-52)	0.006	40 (32.5-51.75)	53 (34-55)	0.040
Sex (*n* (%))				0.522			0.332
Male	50 (60.2%)	23 (65.7%)	21 (58.3%)		45 (62.5%)	5 (45.6%)	
Female	33 (39.8%)	12 (34.3%)	15 (41.7%)		27 (37.5%)	6 (54.5%)	
BMI	24 (21.6-25.6)	24.6 (22.9-26.6)	23.3 (21.2-24.9)	0.028	24.2 (21.8-25.9)	22.4 (20.8-25.4)	0.271
Source of infection (*n* (%))				0.374			0.104
Family cluster	33 (39.8%)	11 (31.4%)	15 (41.7%)		26 (36.1%)	7 (63.6%)	
Imported from Wuhan	50 (60.2%)	24 (68.6%)	21 (58.3%)		46 (63.9%)	4 (36.4%)	
Underlying comorbidity	30 (36.1%)	8 (22.9%)	17 (47.2%)	0.032	26 (36.1%)	4 (36.4%)	1.000
Hypertension	7 (8.5%)	2 (5.7%)	4 (11.1%)	0.414	6 (8.3%)	1 (9.1%)	1.000
Congenital heart disease	1 (1.2%)	0 (0%)	1 (2.8%)	1.000	1 (1.4%)	0	1.000
Diabetes	4 (4.9%)	1 (2.9%)	3 (8.3%)	0.614	4 (5.6%)	0	1.000
Chronic hepatitis	6 (7.3%)	3 (8.6%)	3 (8.3%)	1.000	6 (8.3%)	0	1.000
Chronic lung disease	5 (6.02%)	1 (2.9%)	2 (5.6%)	1	4 (5.6%)	1 (9.1%)	0.518
Postoperative biliary diseases	5 (6.1%)	1 (2.9%)	3 (8.3%)	0.614	4 (5.6%)	1 (9.1%)	0.518
Chronic dyspepsia	3 (3.7%)	1 (2.9%)	2 (5.6%)	1	3 (4.2%)	0	1.000
Cerebrovascular disease	1 (1.2%)	1 (2.9%)	0	0.493	1 (1.4%)	0	1.000
Chronic kidney disease	2 (2.4%)	0	1 (2.8%)	1	1 (1.4%)	1 (9.1%)	0.249
Temperature	37.2 (36.6-37.6)	37.3 (36.8-37.7)	36.8 (36.5-37.4)	0.849	37.3 (36.8-37.8)	36.6 (36.1-37.0)	0.002
Pulse beats per min	90 (81-99)	90 (78-97)	92 (84-100)	0.343	90.5 (81.5-99.8)	84 (73-92)	0.148
Respiratory rate	20 (19-20)	20 (20-20)	20 (19-20)	0.383	20 (19-20)	20 (18-20)	0.314
Systolic blood pressure	126 (116-136)	123 (116-135)	130 (122-140)	0.521	126.5 (116.5-136)	121 (114-141)	0.657
White blood cell count	4.28 (3.54-5.57)	4.28 (3.6-5.03)	4.30 (3.32-5.84)	0.365	4.29 (3.53-5.48)	4.2 (3.56-7.02)	0.638
Lymphocyte count (×10^9^/L)	1.2 (0.9-1.4)	1.2 (0.8-1.4)	1.05 (0.8-1.4)	0.823	1.1 (0.8-1.4)	1.3 (1.0-1.7)	0.205
≥1	57 (68.7%)	26 (74.3%)	20 (55.6%)	0.099	47 (65.3%)	10 (90.9%)	0.160
<1	26 (31.3%)	9 (25.7%)	16 (44.3%)		25 (34.7%)	1 (9.1%)	
Hemoglobin (g/L)	133 (125-145)	135 (126-146)	133 (121-147)	0.817	134 (125.3-146.8)	133 (123-139)	0.582
Platelet count (×10⁹/L)	137 (118-167)	143 (119-167)	140 (118-176)	0.472	137 (118-165)	149 (107-173)	0.586
Total bilirubin (*μ*mol/L)	12.4 (9.8-15.8)	11.8 (9.7-15.2)	12.8 (9.1-15.8)	0.68	12.1 (9.63-15.75)	13 (11.6-17.9)	0.179
ALT (U/L)	29 (18-44)	31 (24-45)	29 (20-48)	0.994	29 (21-44.75)	18 (16-34)	0.057
AST (U/L)	27 (22-38)	29 (22-38)	28 (23-42)	0.391	28.5 (23-38.75)	24 (20-29)	0.105
Albumin (g/L)	39.7 (37.3-41.6)	39.8 (37.8-42.8)	39.8 (36.5-41.4)	0.419	39.8 (37.3-41.58)	38.6 (34.4-42.9)	0.506
<40	39(47%)	18 (51.4%)	14(38.9%)	0.288	32(44.4%)	7(63.6%)	0.235
Lactate dehydrogenase (U/L)	140 (118-177)	143 (116-171)	149 (121-184)	0.25	146.5 (118-178.5)	134 (110-139)	0.227
C-reactive protein (mg/L)	10.4 (3.3-24.4)	11.1 (3.7-24.4)	14.1 (6.3-38.8)	0.097	12.05 (4.15-28)	3.1 (1.8-4.1)	0.001
>6	52 (63%)	22 (62.9%)	28 (77.8%)	0.168	50 (69.4%)	2 (18.2%)	0.002
Procalcitonin (ng/mL)	0.058 (0.043-0.081)	0.058 (0.045-0.081)	0.06 (0.044-0.082)	0.272	0.059 (0.045-0.082)	0.043 (0.038-0.055)	0.019
High-sensitivity cardiac troponin I (pg/mL)	8.5 (6.7-11)	8.05 (5.73-9.55)	7.5 (6.2-10.5)	0.897	7.9 (5.88-10.4)	11.2 (9.35-13.5)	0.008
D-dimer (*μ*g/mL)	0.41 (0.34-0.53)	0.37 (0.34-0.495)	0.42 (0.34-0.54)	0.096	0.41 (0.34-0.53)	0.43 (0.34-0.64)	0.528
>0.5	21 (28.4%)	7 (21.2%)	10 (32.3%)	0.317	17 (26.2%)	4 (40%)	0.452
APTT (s)	40.8 (37.6-44.3)	41.6 (38.2-45.1)	40.7 (37.8-44.4)	0.938	41 (38.1-44.5)	37.2 (36.1-41.5)	0.113
>40	48 (57.8%)	21 (60%)	23 (63.9%)	0.736	44 (61.1%)	4 (36.4%)	0.189
Imaging features				0.547			0.124
Normal	10 (12%)	3 (8.6%)	4 (11.1%)		7 (9.7%)	3 (27.3%)	
Pulmonary infiltration	73 (88%)	32 (91.4%)	32 (88.9%)	1.000	65 (90.3%)	8 (72.7%)	
Bilateral ground-glass opacity	33 (39.8%)	13 (37.1%)	16 (44.4%)		30 (41.7%)	3 (27.3%)	
Local infiltration	15 (18.1%)	7 (20%)	3 (8.3%)		10 (13.9%)	5 (45.5%)	
Multiple patch opacity	25 (30.1%)	12 (34.3%)	13 (36.1%)		25 (34.7%)	0	
Signs and symptoms	72 (86.6%)						
Chills	24 (29.3%)	8 (22.9%)	16 (44.4%)	0.055			
Fever	58 (70.7%)	29 (82.9%)	29 (80.6%)	0.802			
Dry cough	24 (29.3%)	14 (40.0%)	10 (27.8%)	0.276			
Expectoration	23 (28%)	7 (20%)	15 (41.7%)	0.048			
Chest distress	11 (13.4%)	5 (14.3%)	6 (16.7%)	0.782			
Pharyngalgia	9 (11%)	2 (5.7%)	7 (19.4%)	0.151			
Anorexia	10 (12.2%)	5 (10.6%)	5 (14.3%)	0.478			
Headache	6 (7.3%)	3 (8.6%)	3 (8.3%)	1.000			
Dizziness	2 (2.5%)	2 (5.7%)	0	0.239			
Fatigue	29 (35.8%)	11 (32.4%)	18 (50%)	0.134			
Myalgia	18 (22%)	6 (17.1%)	12 (33.3%)	0.117			
Nausea and/or vomiting	5 (6.1%)	1 (2.9%)	3 (8.3%)	0.614			
Diarrhea or bellyache	4 (4.9%)	1 (2.9%)	3 (8.3%)	0.614			
Shapeless stool	4 (4.9%)	2 (5.7%)	1 (2.8%)	0.614			
Disease severity status				0.237			
Asymptomatic	11 (13.3%)						
Mild	71 (85.5%)	35 (49.3%)	36 (50.7%)				
Severe	1 (1.2%)						

Data are median (IQR) or *n* (%), where “*n*” is the number of discharged patients with laboratory-confirmed infections with SARS-CoV-2. *p* values were calculated by the Mann-Whitney *U* test, *χ*^2^ test, or Fisher's exact test, as appropriate. ALT = alanine aminotransferase; AST = aspartate aminotransferase; APTT = activated partial thromboplastin time.

**Table 2:** Treatments and clinical outcomes.

	Total	Mildly symptomatic			Total confirmed SARS-COV-2		
		Viral clearance	Viral persistence	*p* value	Symptomatic	Asymptomatic	*p* value
	*n* = 83	*n* = 35	*n* = 36		*n* = 72	*n* = 11	
Treatments							
Antibiotics	38 (45.8%)	14 (41.2%)	20 (58.8%)	0.190	35 (48.6%)	3 (27.3%)	0.186
Treatments				0.239			0.092
Two medicines	6 (7.2%)	2 (5.7%)	3 (8.3%)		5 (7.0%)	1 (9.0%)	
Two medicines + TCM	70 (84.3%)	33 (94.3%)	30 (83.3%)		63 (87.5%)	7 (63.6%)	
Two medicines+ribavirin+TCM	6 (7.2%)	0	3 (8.3%)		3 (4.2%)	3 (27.3%)	
Lopinavir/ritonavir medicine	81 (97.6%)	35 (100%)	35 (97.2%)	1.000	71 (98.6%)	10 (90.9%)	0.249
Oseltamivir	11 (13.4%)	2 (5.7%)	8 (22.2%)	0.085	10 (13.9%)	1 (9.0%)	1.000
Corticosteroid	23 (27.7%)	8 (22.9%)	12 (33.3%)	0.327	21 (29.2%)	2 (18.2%)	0.719
Intravenous immunoglobulin	6 (7.2%)	3 (8.6%)	2 (5.6%)	0.674	6 (8.3%)	0	1.000
Nasal oxygen therapy	46 (55.4%)	18 (51.4%)	20 (55.6%)	0.727	39 (54.2%)	7 (63.6%)	0.747
TCM	80 (96.4%)	34 (97.1%)	35 (97.2%)	1.000	70 (97.2%)	10 (90.9%)	0.351
Clinical outcomes							
Time from the illness onset to admission	5 (2-7)	3 (2-5)	7 (5-9)	≤0.001			
Time from the illness onset to diagnosis (days)	4.5 (2-7)	3 (1-5)	7 (4-9)	≤0.001			
Time from illness to viral clearance (days)	16 (13-18)	13 (12-14)	18 (17-20.75)	≤0.001			
Duration from illness to radiologic recovery (days)	15 (11-18)	11 (9.25-15)	17 (14-19)	≤0.001			
Duration of lopinavir/ritonavir therapy (days)	10 (10-11)	10 (10-11)	11 (10-11)	0.181	11 (10-11)	10 (6-11)	0.229
Hospital length of stay (days)	16 (14-19)	15 (13-16%)	16 (18-21%)	≤0.001	16 (14-18)	18 (14-22)	0.246

Data are median (IQR) or *n* (%). *p* values were calculated by the Mann-Whitney *U* test, *χ*^2^ test, or Fisher's exact test, as appropriate. Two medicines: INF alpha-2b inhale+lopinavir/ritonavir; TCM: traditional Chinese medicine.

**Table 3:** Influence factors associated with the length of viral clearance (multiple linear regression).

Model	Unstandardized coefficients		Standardized coefficients	*t*	*p*	95% confidence interval for *B*	
	*B*	Std. error	Beta			Lower bound	Upper bound
(Constant)	11.103	1.366		8.127	0.000	8.375	13.831
Corticosteroids^∗^	-0.639	0.931	-0.060	-0.686	0.495	-2.497	1.220
Pneumonia^∗^	-1.343	1.424	-0.083	-0.943	0.349	-4.186	1.500
Delayed admission	1.057	0.124	0.746	8.546	≤0.001	0.810	1.304
Comorbidities	1.907	0.856	0.191	2.228	0.029	0.198	3.616

∗No pneumonia and corticosteroids were control. *R*^2^ = 0.731, adjusted *R*^2^ = 0.534, and Durbin‐Watson = 1.605, adjusted for corticosteroids, pneumonia, delay in admission, and comorbidities. Length of viral clearance = 11.103-0.639 corticosteroids (yes) − 1.343pneumonia (yes) + 1.057 delay in admission + 1.907 comorbidities.

## Data Availability

*Nature of Data*. Our data is from a clinical study and is derived from the electronic medical records of Anqing Municipal Hospital, Anhui, China. *Data Access Restrictions*. The clinical case data used to support the findings of this study are restricted by the Institutional Ethics Board of Anqing Municipal Hospital (no. 2020051) in order to protect patient privacy. Data are available from Mei-man Shao (aqslyy@163.com) for researchers who meet the criteria for access to confidential data. We will provide partial data, which is principal to our manuscript, to such requests. Requests for access to further data should be also made to Mei-man Shao (aqslyy@163.com).
